# Movement Dynamics of Treadmill Running Within the Different Exercise Intensity Domains in Male Runners

**DOI:** 10.1002/ejsc.70236

**Published:** 2026-08-13

**Authors:** Ben Hunter, Bettina Karsten, Mark Burnley, Andrew Greenhalgh, Daniel Muniz‐Pumares

**Affiliations:** ^1^ School of Human Sciences London Metropolitan University London UK; ^2^ School of Health Medicine and Life Sciences University of Hertfordshire Hatfield UK; ^3^ CBS University of Applied Sciences Faculty of Health & Education Cologne Germany; ^4^ Institute of Lifecourse Development, Centre for Exercise Activity and Rehabilitation University of London, University of Greenwich London UK; ^5^ School of Sport Exercise and Health Sciences Loughborough University Loughborough UK

**Keywords:** correlation dimension, endurance running, gait variability, local dynamic stability, lyapunov exponent, non‐linear dynamics

## Abstract

The aetiology of fatigue differs between exercise intensity domains, but limited research has explored non‐linear gait dynamics across them. To address this, this study aimed to investigate movement variability and stability in moderate, heavy, and severe intensity domains, and to examine how these dynamics evolve over time. Twenty‐one male runners performed treadmill trials at speeds corresponding to these domains, with lower limb joint angles and centre of mass displacement recorded using 3D motion analysis and analysed at the start, middle, and end of each trial. Non‐linear dynamics of joint angles and centre of mass displacement including local dynamic stability, dimensionality, and variability were assessed using Lyapunov exponent (LyE), correlation dimension (CoD), and MeanSD, respectively. Repeated‐measures ANOVAs revealed a significant effect of intensity across most parameters, including CoD and LyE at the ankle, knee, and hip (all *p* < 0.05), as well as MeanSD at the hip (*p* < 0.05). Furthermore, a significant effect of intensity was observed in 6 out of the 9 parameters measured at the centre of mass. Effects of time were sparse, with interaction effects largely confined to centre of mass variability. Post hoc analyses indicated that higher intensity domains were associated with reduced local dynamic stability, lower dimensionality, and increased variability at some sites. Changes over time were not exclusive to the severe intensity domain, suggesting that metabolic stress does not play an exclusive role in regulating movement dynamics during running. Collectively, these findings suggest that between‐domain differences in gait dynamics were more consistent with speed‐related task constraints than with cumulative fatigue or metabolic stress alone.

## Highlights

- Running intensity strongly modulates non‐linear gait dynamics, with higher speeds producing greater instability, lower dimensionality, and increased variability across joints and centre of mass.
- Temporal changes in movement dynamics were minimal across all intensity domains, suggesting that speed‐related task constraints may exert a greater influence on non‐linear gait dynamics than cumulative metabolic stress.
- Severe‐intensity running elicited the greatest instability and variability alongside reduced dimensionality, suggesting that neuromuscular control becomes more constrained yet less stable at higher mechanical demands.
- The findings suggest that non‐linear gait metrics are strongly influenced by speed. Therefore, interpretations of fatigue based on non‐linear metrics require careful consideration of task‐related constraints.

## 1 Introduction

Movement variability has historically been viewed as measurement noise that should be eliminated before analysis (Stergiou 2018). As such, single parameters taken from continuous variables, for example, peak angles, have been used to characterise changes to movement strategies (e.g., Apte et al. 2021). Alternatively, techniques such as statistical parametric mapping allow for analysis of entire waveforms (Valencia et al. 2023), but still treat each stride as an independent process. Dynamical systems theory, when applied to gait analysis, suggests that movement patterns arise from a self‐organisation process based on task (e.g., different running speeds (Hunter et al. 2023)), environment (e.g., running surface (Lindsay et al. 2014)), and organismic or individual constraints (e.g., different performance levels (Mo and Chow 2018) or fatigue (Fuller et al. 2017)). This approach allows an exploration of the temporal evolution and interdependence of movement patterns (Stergiou and Decker 2011). Moreover, dynamical systems theory recognises that movement variability is a necessity in gait, allowing individuals to adapt to changes in environment. It has been suggested that a continuum of variability exists, with too much (i.e., unstable) or too little (i.e., rigid) movement variability being maladaptive (Stergiou and Decker 2011). As a result, dynamical systems theory has given rise to the application of non‐linear methods of analysis when analysing gait (Hunter et al. 2023; Stergiou 2018; Strongman and Morrison 2020; Winter et al. 2023).

A multitude of non‐linear methods can be used to assess movement variability, including entropy (Hunter et al. 2021; Yentes et al. 2013), correlation dimension (CoD; Grassberger and Procaccia 1983; Raffalt et al. 2020, 2020b), the Lyapunov exponent (LyE; Raffalt et al. 2019; Rosenstein et al. 1993; Winter et al. 2023), and detrended fluctuation analysis (DFA‐α; Hunter et al. 2021; Jordan et al. 2006; Peng et al. 1994; Phinyomark et al. 2020). The LyE quantifies local dynamic stability, considered as a system's sensitivity to small perturbations, by measuring how neighbouring trajectories diverge over time, and is commonly used to assess control errors during gait (Bruijn et al. 2013; Winter et al. 2023). Although a system is formally stable when LyE ≤ 0, negative exponents are rarely observed in biological locomotion. Healthy human gait typically yields small positive values, so stability is interpreted in relative terms, with greater values indicating faster divergence and a less stable pattern. The CoD estimates the fractal dimension of a system's attractor (Stergiou 2018), with lower values indicating fewer degrees of freedom and a more constrained system, and higher values reflecting greater complexity and adaptability. By retaining the entire time series rather than discrete measures such as knee flexion at initial contact, these methods capture the subtle within‐ and between‐stride fluctuations that yield greater insight into neuromuscular control (Dingwell and Cusumano 2000; Hausdorff et al. 1997). Task constraints have most often been examined by varying running speed (Hunter et al. 2023): movement is typically most stable around the preferred speed (Jordan et al. 2009; Lindsay et al. 2014; Mann et al. 2015; Strongman and Morrison 2021), whereas higher speeds are associated with lower local dynamic stability and greater variability, albeit across speeds that differ considerably between studies (Hunter et al. 2021; Look et al. 2013; Mehdizadeh et al. 2014).

When considering organismic, or individual, constraints, limited research has focussed attention on changes to non‐linear dynamics during running through altering the metabolic environment (Hoenig et al. 2019; Hollander et al. 2021; Hunter et al. 2021; Mo and Chow 2018; Murray et al. 2017). Metabolite accumulation has been postulated to negatively impact stability during quiet standing (Mello et al. 2010) and walking (Vieira et al. 2016) after severe intensity exercise through inhibitory effects on *α*‐motoneurons by activating fatigue‐sensitive muscle afferents. However, conflicting evidence exists, with unchanged dynamics after incremental exercise (Mahaki et al. 2020) and increased stability at the end of a maximal effort 5000‐m run (Hoenig et al. 2019). Exercise intensity can be characterised according to three exercise intensity domains, which each have discrete metabolic and acid‐base responses to exercise (Black et al. 2017; Burnley and Jones 2007). These include the moderate (work rate below the lactate threshold, gas exchange threshold, or ventilatory threshold), heavy (work rate between lactate threshold, gas exchange threshold, or ventilatory threshold and the critical speed), and the severe (work rate above critical speed) (Burnley and Jones 2007). Crucially, the metabolic environment and the mechanical demand of running are difficult to separate entirely: manipulating exercise intensity perturbs the metabolic and acid‐base milieu, and therefore neuromuscular function (Brownstein et al. 2021), while simultaneously altering the mechanical demands of the task, such as stride frequency, ground reaction forces, and joint kinematics (Apte et al. 2021). Examining the mechanical responses during constant work‐rate trials within each domain allows temporal changes in non‐linear dynamics to be examined while running speed remains constant. This provides insight into the influence of the evolving metabolic and acid‐base environment within a fixed speed condition, whilst recognising that between‐domain comparisons necessarily involve different running speeds.

Previous investigations of non‐linear dynamics of running gait (Hunter et al. 2021) and force production (Pethick et al. 2016, 2019) in the heavy and severe intensity domains suggest that motor control strategies may be dependent on the metabolic environment. Only two studies have examined movement dynamics near the boundary of the moderate and heavy intensity domains (Hollander et al. 2021; Mo and Chow 2018). Mo and Chow (2018) found DFA‐α of stride intervals fluctuated during a 31‐min run but reported high blood lactate ([La]) and ratings of perceived exertion values, suggesting intensity exceeded the intended boundary (moderate‐to‐heavy intensity). Hollander et al. (2021) observed increased local dynamic stability over the course of a 15‐min run at 70% of maximal oxygen uptake ($\dot{V}$O_2max_), though the use of a fixed percentage of $\dot{V}$O_2max_ may lead to varied physiological responses (Meyler et al. 2021). To better understand movement dynamics across intensity domains, running speeds should be anchored to physiological thresholds. The aim of this study was consequently to comprehensively examine the temporal dynamics of centre of mass (CoM) displacement and lower limb joint rotations across the three exercise intensity domains. It was hypothesised that running at greater intensities would result in more unstable (evidenced by greater LyE values), variable (evidenced by greater MeanSD values), and complex (evidenced by greater CoD values) temporal dynamics when compared with the other domains. Furthermore, it was hypothesised that changes to temporal dynamics and variability over time would occur exclusively when running in the severe intensity domain, with more unstable and variable running gait towards task failure.

## 2 Methods

### 2.1 Participants

Twenty‐one male runners (age: 32.3 ± 11.2 yr; stature: 1.79 ± 0.07 m; body mass: 74.6 ± 12.1 kg) were recruited to participate after giving written informed consent. A sample size of 17 participants was determined a priori using G*Power (Version 3.1, Bonn University, Germany), based on a repeated‐measures ANOVA (within‐factors). The calculation assumed a medium effect size (*f* = 0.3), an alpha level of 0.05, and a power of 0.80. This effect size was informed by previous work examining sagittal plane hip regularity across exercise intensity domains (Hunter et al. 2021), as the hip is the joint most directly implicated in the propulsive demands that change with running speed (Willer et al. 2024). All participants were in good health and had no known history of serious lower limb injuries which could affect gait. The study was approved by the local ethics committee and adhered to the Declaration of Helsinki except for pre‐registration. All participants were runners or endurance athletes, for example, triathletes, familiar with treadmill running, aged between 18 and 50 years, with a $\dot{V}$O_2max_ ≥ 40 mL·min^−1^ kg^−1^, and a self‐reported 5‐km time of ≤ 24 min or 10‐km time of ≤ 50 min. The level of the participants corresponded to performance level 2 in the classification of participant groups in sport science research (McKay et al. 2021). Exclusion criteria included vestibular or vision disorders, an observed $\dot{V}$O_2max_ < 40 mL·min^−1^ kg^−1^, pulmonary, neurological, or cardiovascular diseases, and current or recent (6 months before participation) musculoskeletal injuries to the lower extremity or back. Assessment of the exclusion criteria, except for $\dot{V}$O_2max_, was carried out by questionnaire during an initial screening on their first visit. Participants were instructed to arrive at the laboratory rested and advised to avoid food in the 3 h before testing, and to avoid strenuous exercise and refrain from alcohol in the 24 h before testing. Caffeine intake before testing was consistent between all visits. All tests were performed at the same time of day (± 2 h) in laboratory conditions within a controlled environment at sea level.

### 2.2 Experimental Design

Participants were required to visit the laboratory on two occasions over a 1‐week period, with visits separated by a minimum of 48 h. All tests were performed on a motorised treadmill (Pulsar, h/p/cosmos Sport and Medical, Traunstein, Germany) set at an incline of 1% (Jones and Doust 1996). The first session was used to familiarise participants with the procedures and to take anthropometric measures including stature and body mass. Following this, a graded exercise test was performed to determine the gas exchange threshold (GET), $\dot{V}$O_2max_, and the speed which evoked GET (sGET) and $\dot{V}$O_2max_ (s$\dot{V}$O_2max_). During the second visit participants ran for 20 min or to task failure, whichever occurred sooner, within three different intensity domains. Lower limb kinematics were recorded throughout the second visit using 3D motion analysis.

### 2.3 Determination of Physiological Thresholds

In the first visit, following the completion of a standardised warm‐up at an intensity of 6 km·h^−1^ for 5 min, the test immediately began at a speed of 8 km·h^−1^ and increased by 0.5 km·h^−1^ every 30 s until volitional exhaustion despite strong verbal encouragement. The speed which elicited s$\dot{V}$O_2max_ was given as the speed of the last stage of the incremental exercise test fully completed. If the final stage was not completed in full, s$\dot{V}$O_2max_ was calculated as: s$\dot{V}$O_2max_ = LCS +(t/(30 s))x 0.5 km·h^1^; where LCS represents the last completed stage (km·h^−1^), t is the time of the incomplete stage performed, 30 s refers to the step duration, and 0.5 km·h^−1^ denotes the delta speed from the previous stage. $\dot{V}$O_2max_ corresponded to the highest $\dot{V}$O_2_ obtained over a 30‐s rolling mean. The sGET was established as the speed that elicited the following criteria: (i) the first disproportionate increase in $\dot{V}$CO_2_ from visual inspection of individual plots of $\dot{V}$CO_2_ vs. $\dot{V}$O_2_; (ii) an increase in $\dot{V}$E/$\dot{V}$O_2_ with no concomitant increase in $\dot{V}$E/$\dot{V}$CO_2_; and (iii) the first increase in end‐tidal O_2_ with no fall in end‐tidal CO_2_ tension (Vanhatalo et al. 2016).

### 2.4 Experimental Trials

In the second visit, runs were performed at speeds corresponding to 80% sGET (moderate (MOD)); and at 20%, and 80% of the difference between sGET and s$\dot{V}$O_2max_ (heavy (HVY) and severe (SEV), respectively). Running trials were performed in a sequential order from lowest intensity to highest, that is, MOD, followed by HVY, and then SEV. The work rates prescribed in the current study are based on previous work to allow for examination of running dynamics in the moderate, heavy, and severe domains (Carter et al. 2002). After the first bout of exercise at 80% sGET, 20‐min rest was permitted before the second bout. A 60‐min period of rest was given between HVY and SEV to allow for adequate recovery and to avoid priming effects (Burnley et al. 2006; Karsten et al. 2017). Participants were allowed to consume water *ad libitum* during rest periods.

### 2.5 Physiological Measures

Throughout both visits, pulmonary gas exchange was collected breath‐by‐breath using an online gas analyser (MetaLyzer 3B, Cortex Biophysik, Leipzig, Germany) and subsequently exported as 10‐s bins following the removal of errant breaths. The amplitude of the $\dot{V}$O_2_ slow component ($\dot{V}$O_2SC_) was calculated as the difference between the 30‐s mean $\dot{V}$O_2_ preceding 3 min and end‐test $\dot{V}$O_2_. Heart rate was recorded throughout each visit beat to beat using a heart rate monitor (Polar H10, Polar Electro, Kempele, Finland). Fingertip capillary blood samples were collected before and after each running bout to assess differences in pre‐ and post‐blood [La] using an automated blood analyser (Biosen C‐Line, EKF Diagnostic, Barleben, Germany).

### 2.6 Motion Analysis

Marker coordinates were captured using a 10‐camera high‐speed motion capture system sampling at 200 Hz throughout each experimental trial (6 Owl cameras and 4 Raptor‐E cameras; Motion Analysis Corp., Santa Rosa, CA). A modified Helen Hayes marker set (Kadaba et al. 1990) was used to place retroreflective markers (diameter: 12 mm) on the skin unilaterally on the first metatarsal, between the second and third metatarsals, fifth metatarsal, calcaneus, lateral malleolus, medial malleolus, lateral femoral epicondyle, medial femoral epicondyle, and bilaterally on the iliac crests, anterior superior iliac spines and sacrum. Clusters of four markers were affixed to the lateral aspect of the shank and thigh and were secured with rigid tape. Definitions for hip, knee, and ankle joint centres remained consistent with the Helen Hayes model (Kadaba et al. 1990), and additional markers were used to enable more consistent marker tracking. Data were inspected using Cortex software (Cortex‐64 6.13.1751, Motion Analysis Corporation, Santa Rosa, CA) before being imported into Visual3D (Visual3D v6x64, C‐motion, Germantown, MD). Static trials were performed immediately before each running trial. From the initial static trials in the anatomical position, kinematic models (pelvis, thigh, shank, and foot) were created for each participant and each trial. Ankle and knee joint centres were defined as the midpoint of the medial and lateral malleolus and the medial and lateral femoral epicondyle markers, respectively. The 3D coordinates of the hip joint centres were approximated using the coordinates of the reflective markers at the left and right anterior superior iliac spines and the marker at the sacrum (Bell et al. 1990). The foot, shank, and thigh were modelled as frusta of right cones, whereas the pelvis was modelled as a cylinder. Segmental anthropometric and geometrical properties were used based on the model by Hanavan (1964). Following the static trials, medial knee and medial ankle markers were removed for subsequent dynamic trials.

### 2.7 Data Analysis

All analyses were performed using custom‐written scripts in MATLAB 2023b (Mathworks, Natick, MA). Marker positional data were down‐sampled to 100 Hz for computational efficiency. Marker data were not filtered prior to analysis based on recommendations by Raffalt et al. (2020b), and to reduce the risk of incorrect identification of ‘noise’, consistent with the approach adopted by previous research (Strongman and Morrison 2021). Similarly, as the same equipment was used for each trial, it was assumed that any noise was consistent between trials, and so any changes between conditions or over time was due to within‐participant variation. Down‐sampling was applied identically across all participants, trials, and intensity domains. Furthermore, state‐space reconstruction and subsequent non‐linear analyses were performed on stride‐normalised data, meaning any residual influence of down‐sampling was common to all conditions. To identify non‐linear dynamics of CoM displacement and lower limb joint angles, the LyE and CoD were calculated. To quantify variability, MeanSD was calculated, where lower values denote lower variability and higher values denote greater variability between strides (James 2004).

First, each experimental trial was separated into epochs of 30 s in length. The minimum number of strides (*n* = 30) observed within a 30‐s epoch across all participants and all trials was selected to ensure consistency between the number of strides analysed (Raffalt et al. 2019). To account for differences in stride time, each variable (CoM displacement and joint angles) was normalised to 101 data points per stride. To calculate normalised stride cycles, foot strikes were determined from the maximum anterior displacement of a heel marker relative to the sacrum marker (Zeni et al. 2008). This method has previously shown close agreement (∼29 ms) with an instrumented treadmill (Fellin et al. 2010). Before calculating LyE and CoD, the CoM displacement and joint angle rotations were reconstructed in state space. In line with previous research (Raffalt et al. 2019), the individual embedding dimension and time delay for each time series were calculated for each participant and each trial using the False Nearest Neighbour (Kennel et al. 1992) and Average Mutual Information (Abarbanel et al. 1993) algorithms, respectively. The LyE was calculated using the Rosenstein et al. (1993) algorithm, with the exponent calculated from the slope of a line fitted to the first stride, and the CoD was calculated in accordance with the methods outlined by Grassberger and Procaccia (1983). Variability (MeanSD) of the CoM displacement and joint angle rotations was calculated in accordance with the methods outlined by Dingwell et al. (2001). After the time series were normalised to 100% of each stride, the standard deviation across all strides (30) was calculated for each timepoint, and the standard deviations across all timepoints were subsequently averaged to give the MeanSD.

### 2.8 Statistical Analysis

All statistical analyses were performed using Jamovi Software (Version 2.3.28.0) and figures drawn in GraphPad Prism (Version 10.1.2). Shapiro‐Wilk tests for normality were conducted on the data. For statistical analysis, three distinct epochs were selected from each trial: the first 30‐s epoch (Start), the epoch corresponding to the midpoint of that participant's trial (Middle), and the final 30‐s epoch (End). A two‐way repeated‐measures analysis of variance was then performed on CoD, LyE, and MeanSD values of the right hip, knee, and ankle joint rotations in the sagittal (flexion/extension) plane of motion, and CoM displacements in mediolateral (ML), vertical (Vert), and anterior‐posterior (AP) directions, to test the effects of exercise intensity domain (MOD, HVY, SEV) and time (Start, Middle, and End). A one‐way analysis of variance was performed to test the effects of intensity (MOD, HVY, SEV) on time to task failure (T_lim_), end pulmonary $\dot{V}$O_2_, $\dot{V}$O_2SC_, and blood [La] responses at the beginning of each trial (baseline) and T_lim_. Differences in $\dot{V}$O_2_ between the initial step test and experimental trials were assessed using a one‐way analysis of variance. Given the number of omnibus tests conducted (54 in total; three effects x three sites x three parameters, for joint angles and centre of mass displacement separately), the false discovery rate was controlled using the Benjamini–Hochberg procedure (Benjamini and Hochberg 1995). Corrections were applied within analysis families defined a priori as each non‐linear parameter (CoD, MeanSD, LyE) within each analysis block (joint angles; centre of mass displacement), giving six families of nine tests. Pairwise comparisons were conducted using Bonferroni adjustments where main effects and interactions were significant once controlled using the Benjamini–Hochberg procedure (*p* < 0.05). Effect sizes were evaluated using partial eta‐squared, with values representing small (ηp^2^ = 0.01), medium (ηp^2^ = 0.06), and large (ηp^2^ = 0.14) effects (Cohen 2013). The assumption of sphericity was tested using Mauchly's test, with Greenhouse‐Geisser corrections made for violations (*p* < 0.05). All data are presented as means ± SD, and results were deemed statistically significant when *p* < 0.05.

## 3 Results

### 3.1 Preliminary Measures and Physiological Responses

The mean sGET and s$\dot{V}$O_2max_ recorded during the first visit were 11.56 ± 0.86 km·h^−1^ and 17.93 ± 1.22 km·h^−1^, respectively. The mean $\dot{V}$O_2max_ observed in the initial step test was 3.81 ± 0.51 L·min^−1^ (52 ± 6 mL·min^−1^ kg^−1^), which was not different from the $\dot{V}$O_2_ observed at T_lim_ in the SEV trials (*p =* 1.000; Table 1). Speed, T_lim_, $\dot{V}$O_2SC_, and physiological variables measured at T_lim_ for each exercise intensity domain are displayed in Table 1.

**TABLE 1:** Trial parameters, pulmonary $\dot{V}$O_2_, blood [La], and heart rate responses during trials performed in the moderate (MOD), heavy (HVY), and severe (SEV) intensity domains.

	MOD	HVY	SEV
Speed (km·h^−1^)	9.25 ± 0.68^a,b^	12.78 ± 0.90^b,c^	16.51 ± 1.15^a,c^
T_lim_ (min)	20.00 ± 0.00^a^	20.00 ± 0.00^b^	4.25 ± 1.20^a,b^
$\dot{V}$O_2_ at T_lim_ (L·min^−1^)	2.41 ± 0.33^a,b^	3.23 ± 0.27^b,c^	3.78 ± 0.50^a,c^
$\dot{V}$O_2SC_ (L·min^−1^)	−0.03 ± 0.18^a,b^	0.17 ± 0.17^b,c^	0.31 ± 0.17^a,c^
Blood [La] at T_lim_ (mmol·L^−1^)	2.33 ± 1.43^a,b^	5.73 ± 3.01^b,c^	10.19 ± 4.00^a,c^
Baseline Blood [La] (mmol·L^−1^)	2.36 ± 1.05	1.76 ± 0.92^a^	2.72 ± 1.44^a^
HR at T_lim_ (bpm)	132 ± 17^a,b^	166 ± 18^b^	170 ± 17^a^

*Note:* Values are expressed as Mean ± SD. Mean values in the same row sharing the same superscript letters are significantly different from each other *p <* 0.05.

Abbreviations: HR, heart rate; T_lim_, time to task failure.

### 3.2 Joint Angles

**FIGURE 1:**
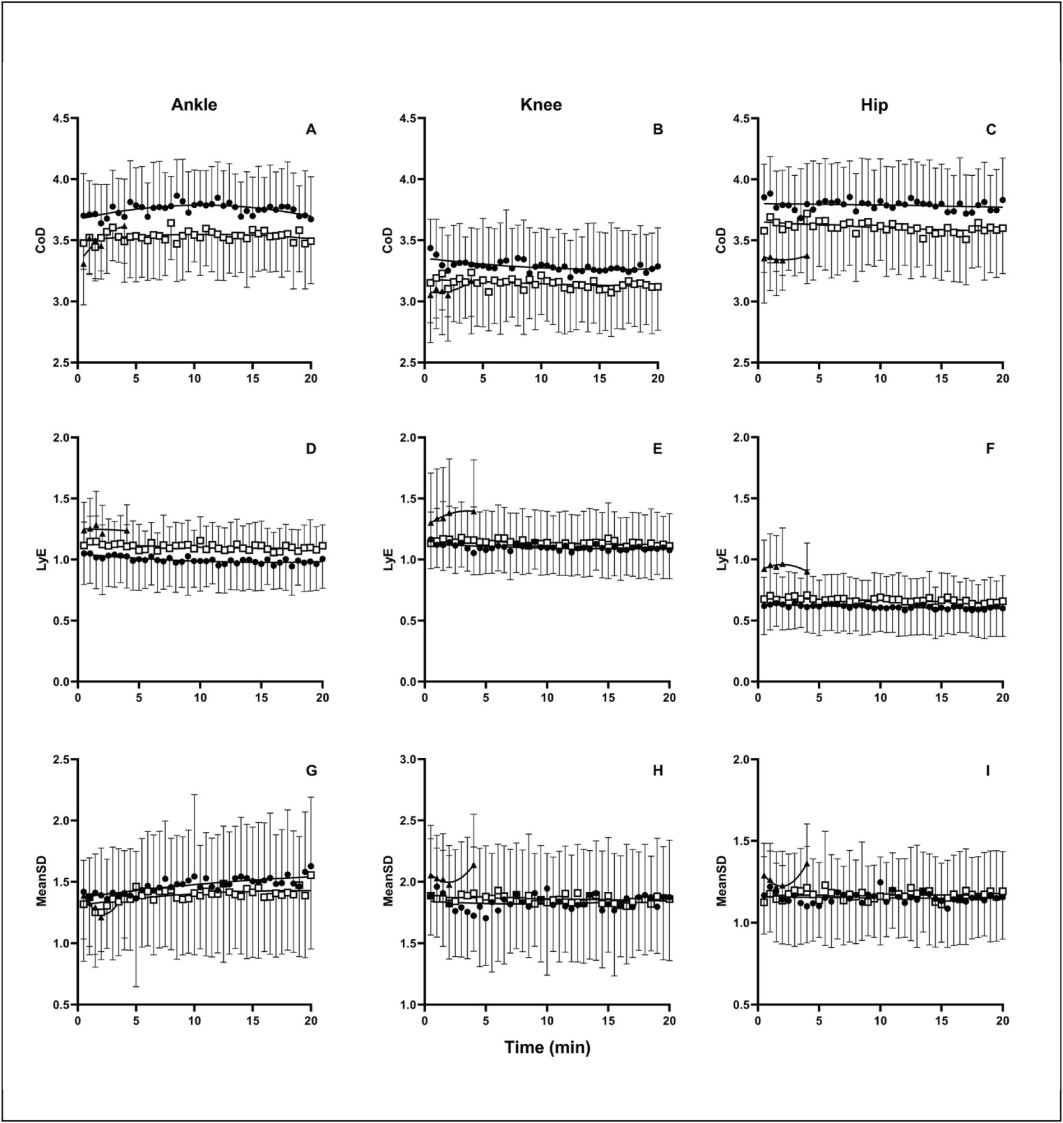
Mean correlation dimension (CoD), Lyapunov exponent (LyE) and MeanSD values for the joint angle data from moderate (black circles), heavy (white squares), and severe (black triangles) trials. Second‐order polynomial curves have been included for illustration. For SEV trials, the mean ± SD values for each epoch completed by all participants, as well as the final epoch, are displayed. Error bars denote SD.

For the ankle, knee, and hip, Intensity effects predominated (Table 2). LyE increased and CoD decreased with higher intensities, with smaller joint‐specific increases in MeanSD (Figure 1). Time effects and Time × Intensity interactions were limited. There was a significant effect of Intensity on LyE for the ankle, knee, and hip (Table 2), with greater values observed at greater intensities (Figure 1). Subsequent post hoc corrections revealed significantly higher LyE values in SEV compared to MOD at the ankle (*p* = 0.004) and hip (*p* < 0.001). Greater LyE values were also observed in SEV compared to HVY at the ankle (*p* = 0.045), knee (*p* = 0.020), and hip (*p* < 0.001). No significant differences in LyE values were observed between MOD and HVY. There were also significant Time × Intensity interaction effects shown for LyE at the knee, with post hoc comparisons demonstrating an increase in LyE from Start to End in SEV (*p* = 0.031), with no further changes observed in other domains.

**TABLE 2:** Primary results for differences between intensity domains (moderate (MOD) versus heavy (HVY) versus severe (SEV)) and time (Start vs. Middle vs. End) in joint angle dynamics. ANOVA results (F‐statistics and *p*‐values) and effect sizes (ηp^2^) are given for main effects of Time and Intensity and for Time × Intensity interaction effects.

Variable	Joint	Domain	Timepoint			ANOVA results		
			Start	Middle	End	Intensity	Time	Time × Intensity
CoD	Ankle	MOD*^#^	3.702 ± 0.346	3.788 ± 0.288	3.672 ± 0.349	**F**_**(2,40)**_ **= 7.63**	F_(1.50,30.04)_ = 3.38	**F**_**(4,80)**_ **= 3.84**
		HVY^#^	3.477 ± 0.222	3.544 ± 0.223	3.493 ± 0.355	***p* = 0.005**	*P* = 0.090	***p* = 0.012**
		SEV*	3.309 ± 0.338	3.479 ± 0.284	3.585 ± 0.306	**ηp** ^**2**^ **= 0.28**	ηp^2^ = 0.14	**ηp** ^**2**^ **= 0.16**
	Knee	MOD*^#^	3.436 ± 0.235	3.301 ± 0.299	3.285 ± 0.319	**F**_**(1.49,29.85)**_ **= 7.04**	F_(1.52,30.36)_ = 0.42	**F**_**(4,80)**_ **= 5.55**
		HVY^#^	3.152 ± 0.333	3.213 ± 0.320	3.120 ± 0.361	***p* = 0.012**	*P* = 0.682	***p* = 0.002**
		SEV*	3.053 ± 0.391	3.071 ± 0.368	3.140 ± 0.433	**ηp** ^**2**^ **= 0.26**	ηp^2^ = 0.02	**ηp** ^**2**^ **= 0.22**
	Hip	MOD*^#^	3.853 ± 0.290	3.822 ± 0.350	3.830 ± 0.343	**F**_**(2,40)**_ **= 37.48**	F_(2,40)_ = 0.27	F_(4,80)_ = 0.82
		HVY^#†^	3.579 ± 0.347	3.618 ± 0.285	3.600 ± 0.375	***p* < 0.001**	*P* = 0.765	*P* = 0.664
		SEV*^†^	3.355 ± 0.367	3.282 ± 0.243	3.353 ± 0.229	**ηp** ^**2**^ **= 0.65**	ηp^2^ = 0.01	ηp^2^ = 0.04
MeanSD	Ankle	MOD	1.418 ± 0.257	1.543 ± 0.670	1.627 ± 0.567	F_(2,40)_ = 2.44	F_(1.38,27.59)_ = 4.54	F_(2.67,53.42)_ = 2.14
		HVY	1.318 ± 0.469	1.408 ± 0.511	1.554 ± 0.608	*P* = 0.169	*P* = 0.094	*P* = 0.113
		SEV	1.387 ± 0.352	1.231 ± 0.291	1.423 ± 0.375	ηp^2^ = 0.11	ηp^2^ = 0.19	ηp^2^ = 0.10
	Knee	MOD	1.883 ± 0.341	1.944 ± 0.705	1.873 ± 0.518	F_(1.63,32.55)_ = 3.68	F_(1.54,30.84)_ = 0.26	F_(4,80)_ = 1.32
		HVY	1.886 ± 0.467	1.831 ± 0.400	1.859 ± 0.480	*P* = 0.100	*P* = 0.715	*P* = 0.347
		SEV	2.052 ± 0.406	1.990 ± 0.381	2.152 ± 0.411	ηp^2^ = 0.16	ηp^2^ = 0.01	ηp^2^ = 0.06
	Hip	MOD	1.167 ± 0.251	1.247 ± 0.383	1.156 ± 0.257	**F**_**(2,40)**_ **= 6.19**	F_(2,40)_ = 0.96	F_(4,80)_ = 2.87
		HVY*	1.125 ± 0.278	1.161 ± 0.245	1.192 ± 0.243	***p* = 0.041**	*P* = 0.440	*P* = 0.094
		SEV*	1.289 ± 0.195	1.227 ± 0.235	1.376 ± 0.242	**ηp** ^**2**^ **= 0.24**	ηp^2^ = 0.05	ηp^2^ = 0.13
LyE	Ankle	MOD*	1.051 ± 0.266	0.988 ± 0.249	1.005 ± 0.241	**F**_**(2,40)**_ **= 9.07**	F_(2,40)_ = 0.26	F_(4,80)_ = 1.43
		HVY^#^	1.114 ± 0.198	1.155 ± 0.208	1.113 ± 0.178	***p* = 0.003**	*P* = 0.970	*P* = 0.417
		SEV*^#^	1.240 ± 0.230	1.261 ± 0.228	1.246 ± 0.209	**ηp** ^**2**^ **= 0.31**	ηp^2^ = 0.01	ηp^2^ = 0.07
	Knee	MOD	1.165 ± 0.272	1.096 ± 0.231	1.077 ± 0.243	**F**_**(1.39,27.74)**_ **= 6.07**	F_(2,40)_ = 0.03	**F**_**(4,80)**_ **= 4.84**
		HVY*	1.132 ± 0.256	1.138 ± 0.257	1.111 ± 0.265	***p* = 0.028**	*P* = 0.970	***p* = 0.005**
		SEV*	1.302 ± 0.407^a^	1.375 ± 0.456	1.403 ± 0.422^a^	**ηp** ^**2**^ **= 0.23**	ηp^2^ = 0.00	**ηp** ^**2**^ **= 0.19**
	Hip	MOD*	0.619 ± 0.236	0.606 ± 0.199	0.600 ± 0.235	**F**_**(2,40)**_ **= 18.35**	F_(2,40)_ = 0.13	F_(4,80)_ = 0.78
		HVY^#^	0.674 ± 0.184	0.690 ± 0.196	0.658 ± 0.215	***p* < 0.001**	*P* = 0.970	*P* = 0.812
		SEV*^#^	0.922 ± 0.236	0.933 ± 0.292	0.946 ± 0.232	**ηp** ^**2**^ **= 0.48**	ηp^2^ = 0.01	ηp^2^ = 0.04

*Note:* Values are expressed as Mean ± SD. Matching symbols denote significant pairwise differences between conditions (*p* < 0.05). Mean values in the same row sharing the same superscript letters are significantly different from each other (*p* < 0.05) between timepoints within condition. Benjamini–Hochberg adjusted *p*‐values displayed to account for false discovery rate. Significant ANOVA results are given in bold (*p* < 0.05).

A significant effect of Intensity was shown on CoD for the ankle, knee, and hip (Table 2; Figure 1). Post hoc corrections showed significant differences between MOD and SEV at the ankle (*p* = 0.012), knee (*p* = 0.018), and hip (*p* < 0.001), with lower values observed at greater intensities. Additionally, significantly lower CoD was observed in HVY relative to MOD at the ankle (*p* = 0.023), knee (*p* = 0.003), and hip (*p* < 0.001). Lower CoD values at the hip were also observed in SEV compared to HVY (*p* < 0.001). When considering the Time × Intensity interactions, there were no significant differences within trials observed once applying post hoc corrections at the knee or ankle.

There was only one significant effect of Intensity on MeanSD at the hip (*p* = 0.041; Table 2), with post hoc analysis demonstrating greater variability in SEV compared to HVY (*p* = 0.006). However, there were no significant effects of Time or Time × Intensity interaction effects.

### 3.3 Centre of Mass Displacement

**FIGURE 2:**
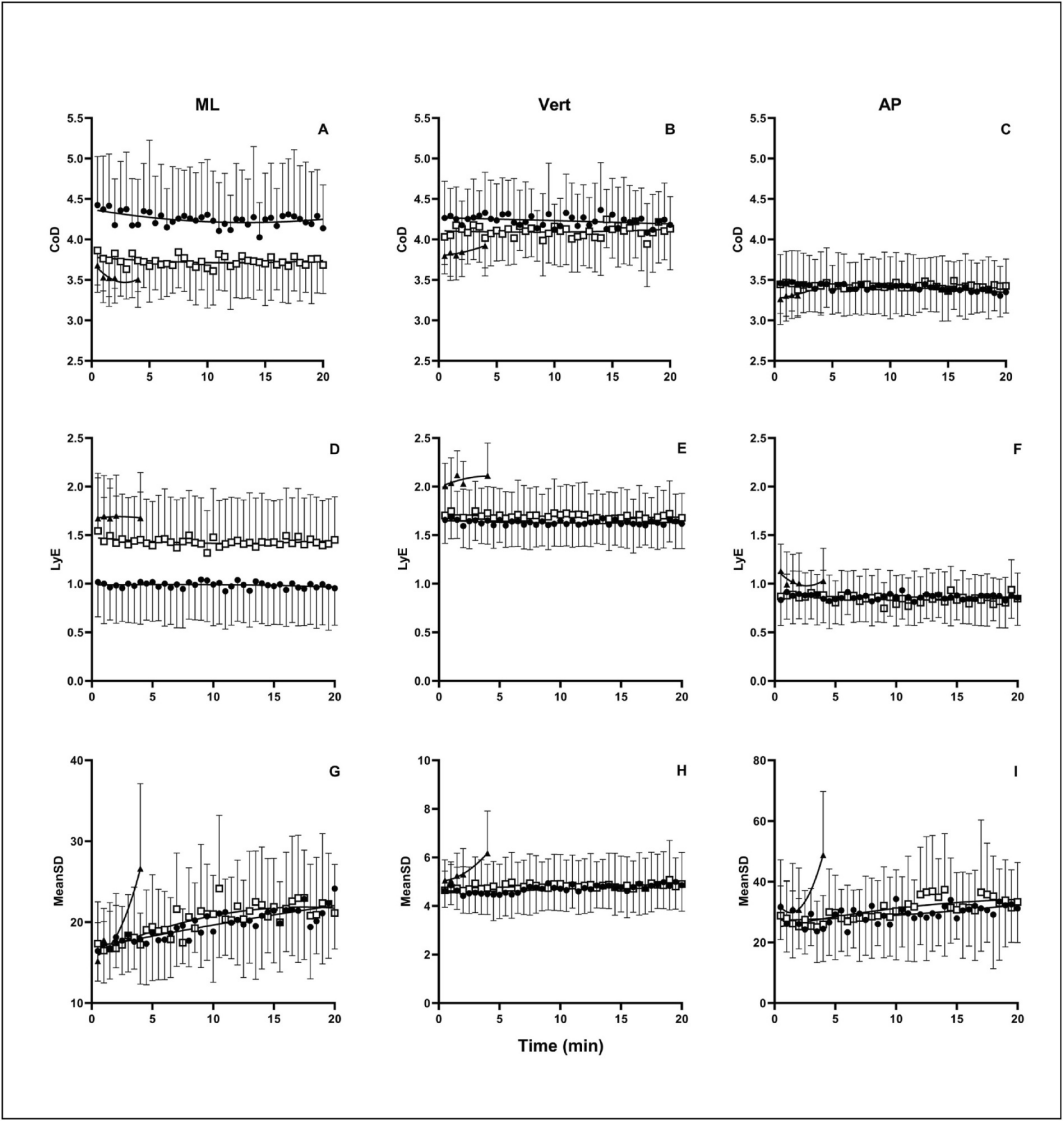
Mean correlation dimension (CoD), Lyapunov exponent (LyE) and MeanSD values for centre of mass displacement in mediolateral (ML), vertical (Vert), and anterior‐posterior (AP) directions from moderate (black circles), heavy (white squares), and severe (black triangles) trials. Second‐order polynomial curves have been included for illustration. For SEV trials, the mean ± SD values for each epoch completed by all participants, as well as the final epoch, are displayed. Error bars denote SD.

A similar pattern emerged for CoM displacement where LyE increased with Intensity across ML and Vert directions, CoD tended to be lower at higher intensities, and variability increased modestly over time in ML and Vert, with interaction effects also evident. There was a significant effect of Intensity on LyE of the CoM displacement in the ML, Vert, and AP directions, with greater values observed at greater intensities (Table 3; Figure 2). Post hoc corrections showed greater LyE in SEV compared to HVY in Vert (*p* < 0.001), as well as in SEV compared to MOD in the ML (*p* < 0.001) and Vert (*p* < 0.001) directions. A significant difference between MOD and HVY was noted in the ML direction (*p* < 0.001). No effects of Time and no interaction effects between Time and Intensity were evident in LyE of CoM displacement.

**TABLE 3:** Primary results for differences between intensity domains (moderate (MOD) versus heavy (HVY) versus severe (SEV)) and time (Start vs. Middle vs. End) in centre of mass displacement. ANOVA results (F‐statistics and *p*‐values) and effect sizes (ηp^2^) are given for main effects of Time and Intensity and for Time × Intensity interaction effects.

Variable	Direction	Domain	Timepoint			ANOVA results		
			Start	Middle	End	Intensity	Time	Time × Intensity
CoD	ML	MOD*	4.424 ± 0.612	4.306 ± 0.710	4.139 ± 0.556	**F**_**(1.59,31.89)**_ **= 31.46**	**F**_**(2,40)**_ **= 12.10**	F_(4,80)_ = 0.85
		HVY^#^	3.864 ± 0.446	3.644 ± 0.504	3.685 ± 0.372	***p* < 0.001**	***p* < 0.001**	*P* = 0.640
		SEV*^#^	3.681 ± 0.333	3.482 ± 0.316	3.472 ± 0.344	**ηp** ^**2**^ **= 0.61**	**ηp** ^**2**^ **= 0.38**	ηp^2^ = 0.04
	Vert	MOD	3.459 ± 0.354	3.416 ± 0.373	3.349 ± 0.409	F_(2,40)_ = 1.58	F_(1.33,26.64)_ = 0.05	**F**_**(4,80)**_ **= 3.60**
		HVY	3.445 ± 0.359	3.423 ± 0.404	3.426 ± 0.337	*P* = 0.333	*P* = 0.887	***p* = 0.021**
		SEV	3.264 ± 0.314	3.301 ± 0.284	3.396 ± 0.324	ηp^2^ = 0.07	ηp^2^ = 0.00	**ηp** ^**2**^ **= 0.15**
	AP	MOD*	4.266 ± 0.456	4.122 ± 0.353	4.180 ± 0.370	**F**_**(2,40)**_ **= 14.50**	F_(2,40)_ = 0.44	F_(4,80)_ = 1.46
		HVY^#^	4.031 ± 0.367	4.161 ± 0.540	4.133 ± 0.530	***p* < 0.001**	*P* = 0.728	*P* = 0.333
		SEV*^#^	3.797 ± 0.223	3.763 ± 0.270	3.916 ± 0.373	**ηp** ^**2**^ **= 0.42**	ηp^2^ = 0.02	ηp^2^ = 0.07
MeanSD	ML	MOD	0.0164 ± 0.0038^a^	0.0188 ± 0.0063	0.0241 ± 0.0076^a^	F_(2,40)_ = 0.05	**F**_**(2,40)**_ **= 34.56**	**F**_**(4,80)**_ **= 3.22**
		HVY	0.0173 ± 0.0053	0.0210 ± 0.0048	0.0211 ± 0.0060	*P* = 0.951	***p* < 0.001**	***p* = 0.025**
		SEV	0.0152 ± 0.0047^a^	0.0190 ± 0.0043	0.0262 ± 0.0105^a^	ηp^2^ = 0.00	**ηp** ^**2**^ **= 0.63**	**ηp** ^**2**^ **= 0.14**
	Vert	MOD*	0.0046 ± 0.0007	0.0047 ± 0.0009	0.0049 ± 0.0011	**F**_**(2,40)**_ **= 21.06**	**F**_**(1.37,27.33)**_ **= 7.18**	**F**_**(4,80)**_ **= 4.05**
		HVY^#^	0.0046 ± 0.0008	0.0049 ± 0.0012	0.0049 ± 0.0013	***p* < 0.001**	***p* = 0.016**	***p* = 0.015**
		SEV*^#^	0.0051 ± 0.0008	0.0052 ± 0.0009	0.0062 ± 0.0017	**ηp** ^**2**^ **= 0.51**	**ηp** ^**2**^ **= 0.26**	**ηp** ^**2**^ **= 0.17**
	AP	MOD*	0.0316 ± 0.0107	0.0343 ± 0.0204	0.0312 ± 0.0114	**F**_**(2,40)**_ **= 5.25**	**F**_**(2,40)**_ **= 3.42**	**F**_**(4,80)**_ **= 3.12**
		HVY	0.0288 ± 0.0101	0.0303 ± 0.0137	0.0333 ± 0.0131	***p* = 0.017**	***p* = 0.048**	***p* = 0.025**
		SEV*	0.0324 ± 0.0149	0.0332 ± 0.0127	0.0480 ± 0.0209	**ηp** ^**2**^ **= 0.21**	**ηp** ^**2**^ **= 0.15**	**ηp** ^**2**^ **= 0.13**
LyE	ML	MOD^#†^	1.017 ± 0.378	0.993 ± 0.440	0.954 ± 0.410	**F**_**(2,40)**_ **= 32.15**	F_(2,40)_ = 1.72	F_(4,80)_ = 0.66
		HVY*^†^	1.544 ± 0.554	1.478 ± 0.516	1.452 ± 0.483	***p* < 0.001**	*P* = 0.223	*P* = 0.622
		SEV*^#^	1.674 ± 0.462	1.678 ± 0.379	1.681 ± 0.469	**ηp** ^**2**^ **= 0.62**	ηp^2^ = 0.08	ηp^2^ = 0.03
	Vert	MOD^#^	1.657 ± 0.290	1.649 ± 0.275	1.619 ± 0.272	**F**_**(2,40)**_ **= 66.80**	F_(2,40)_ = 1.69	F_(2.63,52.67)_ = 1.63
		HVY*	1.703 ± 0.292	1.710 ± 0.296	1.680 ± 0.254	***p* < 0.001**	*P* = 0.223	*P* = 0.223
		SEV*^#^	2.006 ± 0.235	2.105 ± 0.237	2.097 ± 0.335	**ηp** ^**2**^ **= 0.77**	ηp^2^ = 0.08	ηp^2^ = 0.08
	AP	MOD	0.833 ± 0.265	0.865 ± 0.299	0.857 ± 0.289	F_(2,40)_ = 4.24	F_(2,40)_ = 2.75	F_(4,80)_ = 2.18
		HVY	0.869 ± 0.248	0.791 ± 0.293	0.847 ± 0.271	*p* = 0.064	*P* = 0.142	*P* = 0.142
		SEV	1.132 ± 0.275	1.006 ± 0.292	0.972 ± 0.335	ηp^2^ = 0.17	ηp^2^ = 0.12	ηp^2^ = 0.10

*Note:* Values are expressed as Mean ± SD. Matching symbols denote significant pairwise differences between conditions (*p* < 0.05). Mean values in the same row sharing the same superscript letters are significantly different from each other (*p* < 0.05) between timepoints within condition. Benjamini–Hochberg adjusted *p*‐values displayed to account for false discovery rate. Significant ANOVA results are given in bold (*p* < 0.05).

For the CoD of CoM displacement, there were significant effects of Intensity in the ML and AP directions (Table 3), with greater CoD values typically shown at lower intensities (Figure 2). Post hoc comparisons showed CoD values in MOD were higher than HVY (*p* < 0.001) and SEV (*p* < 0.001) in the ML direction and lower CoD values in SEV compared to HVY (*P* = 0.003) and MOD (*p* < 0.001) in the AP direction. There was a significant effect of Time on the CoD of CoM displacement in ML (Table 3; Figure 2) with a decrease in CoD from the Start to Middle (*p* = 0.009) and End (*p* < 0.001). In the Vert direction, there was a significant Time × Intensity interaction, but no significant differences were evident when applying post hoc correction.

When considering variability, there were significant effects of Time and Time × Intensity interaction effects in all directions (Table 3; Figure 2). MeanSD increased from the Start to the Middle (*p* < 0.001) and End (*p* < 0.001) in the ML direction. Similar observations were made in the Vert direction with increased MeanSD observed in the Middle (*p* < 0.001) and End (*p* < 0.001) compared to the Start. No significant differences between timepoints were noted in the AP direction following post hoc corrections. When considering significant interaction effects, there were significant increases from Start to End in the ML (*p* = 0.005) direction in SEV, as well as in MOD, with MeanSD increasing from Start to End (*p* = 0.002). A significant effect of Intensity was evident (Table 3), with significantly greater MeanSD demonstrated in SEV compared to HVY in Vert (*p* < 0.001) and AP (*p* = 0.007) directions, as well as between SEV and MOD in the Vert direction (*p* < 0.001).

## 4 Discussion

This study sought to investigate the effects of exercise intensity domain on the temporal dynamics of CoM displacement and lower limb joint rotations during running. The physiological responses presented in Table 1 demonstrate the prescribed speeds were appropriate to ensure participants were within the three intensity domains. Moreover, $\dot{V}$O_2_ at T_lim_ in the SEV trial was not different from the $\dot{V}$O_2max_ attained during the initial graded exercise test. Pulmonary $\dot{V}$O_2_ in the moderate intensity domain achieved a steady‐state within 3 min, confirmed by visual inspection of the data. In partial support of our hypotheses, the findings demonstrate that movement variability and stability are modulated by exercise intensity, with the severe domain eliciting greater instability and variability compared to the moderate and heavy domains. However, the temporal evolution of movement dynamics over time was not observed exclusively in the SEV trial, suggesting that changes to non‐linear movement dynamics were more consistent with speed‐related task constraints than with cumulative metabolic stress. Therefore, our secondary hypothesis is refuted.

In both HVY and SEV trials, LyE was higher compared to MOD, suggesting greater instability, and CoD lower, indicating a more deterministic structure, at greater intensities. Furthermore, most of these differences occurred within the severe intensity domain when compared to lower intensities. This is in accordance with earlier work that demonstrated distinct torque regularity and variability profiles above the critical torque, analogous to the critical speed (Pethick et al. 2016). Similarly, Murray et al. (2017) demonstrated that metabolic rate, inferred from Blood [La], and movement regularity, quantified by accelerometers at the tibia and pelvis, showed significant positive correlations. Hunter et al. (2021) also showed differences in the regularity and fractal dimension of joint angles between the heavy and severe intensity domains. However, in the current study minimal changes in the non‐linear dynamics were evident over time, and few of these occurred only in the severe domain. This is unlike the findings of Pethick et al. (2016) where a progressive change over time in regularity and self‐similarity of torque was observed above the critical torque, suggesting metabolic stress is a key mediator of force production during isolated movements. The differences between the findings of Pethick et al. (2016, 2019) and the present work may be explained by the type of activity being examined. Task failure in isolated movements was given as the inability to meet the target torque on three occasions (Pethick et al. 2016, 2019). However, in the current study task failure in SEV trials coincided with the participants stopping completely, potentially limiting our ability to assess non‐linear dynamics closer to the point of task failure. Indeed, when considering severe intensity running performed overground, during 5 km (Hoenig et al. 2019) and 3.2 km (Schütte et al. 2015) time trials, significant changes in non‐linear dynamics do occur. The behaviour of isolated muscle actions has also been shown to differ from whole body movements under different task constraints (Santuz et al. 2018; Walsh 2021), whereby whole body movement becomes more erratic, but motor synergies become less complex during challenging locomotion.

The results of this study suggest that variability and non‐linear dynamics of movement during treadmill running are more strongly mediated by task constraints, that is, speed, when compared to organismic constraints, that is, metabolic environment, as reported previously (Hunter et al. 2021). Both moderate and heavy intensity domains are characterised by the attainment of a steady state, albeit delayed because of the emergence of the $\dot{V}$O_2SC_ in heavy intensity exercise (Burnley and Jones 2007). Time to task failure in the moderate domain (attributed primarily to glycogen depletion) and heavy domain (attributed mainly to muscle metabolic perturbation and glycogen depletion) has been reported to be more than 200 and 40 min, respectively (Black et al. 2017). Although the mechanisms of fatigue in both domains differ, it is unlikely that these would have had a substantial impact over the course of the 20‐min trials. Despite this, some differences in non‐linear dynamics and variability were observed between MOD and HVY conditions. Given the limited time of fatigue‐related processes to manifest across both conditions, it is suggested that non‐linear dynamics of running gait are affected by running speed, consistent with previous work (Jordan et al. 2009; Look et al. 2013; Mann et al. 2015; Mehdizadeh et al. 2014; Raffalt, Kent, et al. 2020a). Other studies have shown no difference when running speed was altered by 10% (Lindsay et al. 2014; Strongman and Morrison 2021). A 10% manipulation may fall within the range over which runners preserve dynamic similarity, allowing the motor system to accommodate the change without a detectable reorganisation of movement dynamics. Indeed, Strongman and Morrison (2021) argued that anatomically scaled speeds yield more reproducible non‐linear measures. By contrast, the differences between domains in the present study were considerably larger (∼3.7 km·h^−1^ between the heavy and severe trials), which may exceed the threshold at which a comparable control solution can be maintained. Differences in the variable analysed may also contribute, given that stride timing (Lindsay et al. 2014) and joint‐angle measures need not display equivalent sensitivity to speed‐related change.

With increased running speeds, LyE was shown to increase alongside a decrease in CoD. Previous research has shown that CoD *and* LyE of joint angles decrease concomitantly in a curvilinear manner with increasing running speeds (Raffalt, Kent, et al. 2020a). Despite a range of speeds being considered, the highest speed used by Raffalt, Kent, et al. (2020); (2020b) was 12.0 km·h^−1^ due to inexperience of the participants with treadmills. It was posited by the authors that attractor stability would eventually decrease at higher running speeds. Speeds observed in the current study were higher in both HVY (12.8 ± 0.9 km·h^−1^) and SEV (16.5 ± 1.2 km·h^−1^), which resulted in increases in LyE, but a decrease in CoD. The greater instability, that is, higher LyE, coupled with a more deterministic structure, that is, lower CoD, would initially seem to be counterintuitive. When considering the state space, this means that at higher running speeds the system was comprised of fewer dimensions, but the neighbouring trajectories diverge more rapidly. The reduced dimensionality of the system may be a compensatory mechanism adopted by the neuromuscular system to cope with instability. When considering challenges to dynamic stability during locomotion across uneven terrain, a transition to more robust muscle activation patterns was evident alongside diminished local dynamic stability (Santuz et al. 2018). Similar findings were demonstrated when examining muscle activations through a range of running speeds (Santuz et al. 2020). As running speed increased, muscle activation patterns became less complex, suggested to make the successful execution of motor patterns less prone to the influence of external perturbations (Mileti et al. 2020; Santuz et al. 2018). In the context of the current study, it is proposed that the reduced dimensionality of joint angles and CoM displacements may render the runner more robust in the face of increased instability. Indeed, it has been shown previously that the nervous system responds to instability by reducing dimensionality of motor control in participants on unstable surfaces or with cerebral ataxia (Martino et al. 2015). Exploratory work using electromyography may be necessary to explore this further in the context of running within different physiological intensities.

The lower CoD values observed at greater intensities could also be explained through changes to the control mechanisms associated with running at higher speeds. It has been suggested previously that increasing speed beyond the preferred running speed reduces the number of dynamical degrees of freedom (Jordan et al. 2007). When running at higher speeds, there is an increased mechanical demand placed on the body (Saibene and Minetti 2003). To accommodate this, and to ensure efficiency, the body relies on greater stiffness through greater muscle co‐contraction and pre‐activation of muscles before ground contact (Arampatzis et al. 1999; Wilson and Flanagan 2008). As these mechanical and neuromuscular adjustments occur, running movement becomes more constrained, effectively reducing the degrees of freedom available for coordination. Furthermore, at higher running speeds there are a reduced number of viable solutions to navigate the environment. In the current study, it is posited that the simplification of control mechanisms responsible for forward propulsion coupled with a reduced time to explore different movement solutions (Santuz et al. 2020), resulted in a reduction in the dimensionality of the attractor at higher speeds, and therefore a lower CoD.

Previously, local dynamic stability has been shown to decrease at greater walking (Dingwell and Marin 2006; England and Granata 2007) and running speeds (Look et al. 2013; Mehdizadeh et al. 2014), although this has not consistently been demonstrated during running (Mehdizadeh et al. 2016; Strongman and Morrison 2021). These discrepancies may reflect methodological differences between studies, including the magnitude of the speed manipulation, the joint or body segment examined, and whether short‐ or long‐term divergence exponents were computed. For instance, Mehdizadeh et al. (2014) reported that increasing speed reduced local dynamic stability in the short term but left long‐term stability unaffected, illustrating how the choice of measure can determine whether a speed effect is detected. Padulo et al. (2022) examined the effects of different gradients, thereby altering metabolic demand, at the same running speed. Greater stride‐interval variability was associated with the highest metabolic demand, whereas local dynamic stability was influenced primarily by gradient. This differs from the present findings but may reflect methodological differences. Their study analysed stride‐interval time series, whereas we examined the state‐space dynamics of joint angles and centre of mass displacement. In addition, gradient was held constant throughout the current study, removing a factor that appeared to influence local dynamic stability in their work. Further to changes in gradient, movement variability has been shown to increase when running at faster speeds (Hunter et al. 2021; Jordan et al. 2009). Shorter stride durations may limit the time permitted for fine adjustments to accommodate mechanical disturbances or error (England and Granata 2007; Mehdizadeh et al. 2014). Furthermore, greater running speeds increase segmental momentum and ground reaction forces, necessitating greater neural control to mitigate kinematic disturbances. In the current study, given the reduced dimensionality of joint angles and CoM displacement, it is posited that these mechanical disturbances were not attenuated, leading to a less stable and more variable gait pattern at higher speeds. Several alternative explanations warrant consideration. Stride frequency necessarily increases with running speed. However, all time series were normalised to 101 points per stride and a fixed number of strides (30) was analysed, so that LyE and CoD are expressed per stride cycle rather than per unit time, and the between‐domain differences cannot be an artefact of differing stride durations. Foot strike pattern was not monitored, and a shift in landing type across the range of speeds studied would carry both mechanical and metabolic consequences (Ardigò et al. 1995). This remains a plausible alternative mechanical explanation for part of the intensity effect, and may be the subject of further research in this area.

### 4.1 Limitations

A limitation in the study design is the fixed trial order, which may have introduced order effects, fatigue effects, and/or changes in motivation. However, baseline blood [La] was only different between heavy and severe trials so should be considered when interpreting the results. In the current study, a motorised treadmill was used to maintain a controlled environment between testing sessions and to enable the collection of large kinematic datasets using 3D motion analysis. When compared to overground running, treadmill running changes the non‐linear dynamics of running gait and muscle activation patterns (Mileti et al. 2020), and so may not be representative of dynamics which occur during overground running. The method of stride detection chosen to normalise the strides may have been affected by changes between rearfoot and forefoot striking patterns, which was not monitored during the study. Additionally, our marker set was confined to the pelvis and lower limbs, and upper‐body contributions to whole‐body angular momentum, potentially relevant to the regulation of mediolateral centre of mass displacement, could not be quantified. The relatively wide age range (range: 19–50 yr) may have introduced some heterogeneity in movement dynamics. Although typically studied in older (> 65 yr) populations, previous research indicates that age can influence gait variability (Mehdizadeh 2018). While all participants were endurance‐trained, we cannot exclude the possibility that age‐related differences may have contributed to heterogeneity in our results. Thirty strides were used to quantify CoD and LyE, which resulted in a shorter time series than sometimes observed in this type of analysis (Winter et al. 2023), and is on the lower end of what is considered robust for CoD estimation. This was chosen based on the minimum number of strides observed across a 30‐s epoch for all participants to allow for the same number of strides and data points to be used. Thirty cycles have been used previously to examine motion capture data of cycling (Abbasi et al. 2019), walking (England and Granata 2007), running (Ogaya et al. 2021), and skiing (Cignetti et al. 2009). The 30‐s period was chosen to allow for an examination of non‐linear dynamics and variability close to the point of task failure, particularly in SEV (T_lim_ range: 2.41–7.20 min), to fulfil the aim of examining temporal dynamics over time. Participants were male, endurance‐trained recreational runners, which limits generalisability to females, elite populations, and overground conditions. Finally, the study design altered exercise intensity through the manipulation of running speed. Further studies should seek to utilise methods which are able to better differentiate between the effects of speed and intensity.

## 5 Conclusion

This study investigated the effects of exercise intensity domains on non‐linear dynamics and variability of CoM displacement and lower limb joint rotations during running. Our findings demonstrate that non‐linear dynamics and variability are influenced by running intensity, with the severe intensity domain eliciting more pronounced changes when compared to lower intensities. However, contrary to our hypothesis, temporal changes in movement dynamics were not observed exclusively during severe‐intensity running. These results suggest that task constraints associated with running speed may play a more dominant role than cumulative metabolic stress in shaping non‐linear movement dynamics during treadmill running. Specifically, more constrained but less stable and more variable movement patterns are observed with increased running speed. Consequently, non‐linear measures of variability, stability, and dimensionality should not be interpreted as direct markers of fatigue without accounting for task constraints. For instance, wearable technology used to monitor gait dynamics during training or competition may capture speed‐induced changes in movement regulation rather than true fatigue‐related effects. The observed adaptations at higher speeds may reflect a robustness‐oriented control strategy to meet increased mechanical demands, although further research is needed to determine whether this is beneficial or detrimental for injury risk and performance.

## Funding

The authors have nothing to report.

## Conflicts of Interest

The authors declare no conflicts of interest.

## Data Availability

The data that support the findings of this study are available from the corresponding author upon reasonable request.

## References

[ejsc70236-bib-0001] Abarbanel, H. D. I. , R. Brown , J. J. Sidorowich , and L. S. Tsimring . 1993. “The Analysis of Observed Chaotic Data in Physical Systems.” Reviews of Modern Physics 65, no. 4: 1331–1392. 10.1103/RevModPhys.65.1331.

[ejsc70236-bib-0002] Abbasi, A. , M. Zamanian , and Z. Svoboda . 2019. “Nonlinear Approach to Study the Acute Effects of Static and Dynamic Stretching on Local Dynamic Stability in Lower Extremity Joint Kinematics and Muscular Activity During Pedalling.” Human Movement Science 66: 440–448. 10.1016/J.HUMOV.2019.05.025. 31176255

[ejsc70236-bib-0003] Apte, S. , G. Prigent , T. Stöggl , et al. 2021. “Biomechanical Response of the Lower Extremity to Running‐Induced Acute Fatigue: A Systematic Review.” Frontiers in Physiology, August 12: 1–16. 10.3389/fphys.2021.646042. PMC8430259 34512370

[ejsc70236-bib-0004] Arampatzis, A. , G. P. Brüggemann , and V. Metzler . 1999. “The Effect of Speed on Leg Stiffness and Joint Kinetics in Human Running.” Journal of Biomechanics 32, no. 12: 1349–1353. 10.1016/S0021-9290(99)00133-5. 10569714

[ejsc70236-bib-0005] Ardigò, L. P. , C. Lafortuna , A. E. Minetti , P. Mognoni , and F. Saibene . 1995. “Metabolic and Mechanical Aspects of Foot Landing Type, Forefoot and Rearfoot Strike, in Human Running.” Acta Physiologica Scandinavica 155, no. 1: 17–22. 10.1111/J.1748-1716.1995.TB09943.X. 8553873

[ejsc70236-bib-0006] Bell, A. L. , D. R. Pedersen , and R. A. Brand . 1990. “A Comparison of the Accuracy of Several Hip Center Location Prediction Methods.” Journal of Biomechanics 23, no. 6: 617–621. 10.1016/0021-9290(90)90054-7. 2341423

[ejsc70236-bib-0007] Benjamini, Y. , and Y. Hochberg . 1995. “Controlling the False Discovery Rate: A Practical and Powerful Approach to Multiple Testing.” Journal of the Royal Statistical Society: Series B 57, no. 1: 289–300. 10.1111/J.2517-6161.1995.TB02031.X.

[ejsc70236-bib-0008] Black, M. I. , A. M. Jones , J. R. Blackwell , et al. 2017. “Muscle Metabolic and Neuromuscular Determinants of Fatigue During Cycling in Different Exercise Intensity Domains.” Journal of Applied Physiology 122, no. 3: 446–459. 10.1152/japplphysiol.00942.2016. 28008101 PMC5429469

[ejsc70236-bib-0009] Brownstein, C. G. , G. Y. Millet , and K. Thomas . 2021. “Neuromuscular Responses to Fatiguing Locomotor Exercise.” Acta Physiologica 231, no. 2: e13533. 10.1111/apha.13533. 32627930

[ejsc70236-bib-0010] Bruijn, S. M. , O. G. Meijer , P. J. Beek , and J. H. Van Dieen . 2013. “Assessing the Stability of Human Locomotion: A Review of Current Measures.” Journal of the Royal Society Interface 10, no. 83: 20120999. 10.1098/rsif.2012.0999. 23516062 PMC3645408

[ejsc70236-bib-0011] Burnley, M. , J. H. Doust , and A. M. Jones . 2006. “Time Required for the Restoration of Normal Heavy Exercise VO_2_ Kinetics Following Prior Heavy Exercise.” Journal of Applied Physiology 101, no. 101: 1320–1327. 10.1152/japplphysiol.00475.2006. 16857864

[ejsc70236-bib-0012] Burnley, M. , and A. M. Jones . 2007. “Oxygen Uptake Kinetics as a Determinant of Sports Performance.” European Journal of Sport Science 7, no. 2: 63–79. 10.1080/17461390701456148.

[ejsc70236-bib-0013] Carter, H. , J. S. M. Pringle , A. M. Jones , and J. H. Doust . 2002. “Oxygen Uptake Kinetics During Treadmill Running Across Exercise Intensity Domains.” European Journal of Applied Physiology 86, no. 4: 347–354. 10.1007/s00421-001-0556-2. 11990749

[ejsc70236-bib-0014] Cignetti, F. , F. Schena , and A. Rouard . 2009. “Effects of Fatigue on Inter‐Cycle Variability in Cross‐Country Skiing.” Journal of Biomechanics 42, no. 10: 1452–1459. 10.1016/j.jbiomech.2009.04.012. 19446817

[ejsc70236-bib-0015] Cohen, J. 2013. Statistical Power Analysis for the Behavioral Sciences, 1–567. 10.4324/9780203771587.

[ejsc70236-bib-0016] Dingwell, J. B. , and J. P. Cusumano . 2000. “Nonlinear Time Series Analysis of Normal and Pathological Human Walking.” Chaos: An Interdisciplinary Journal of Nonlinear Science 10, no. 4: 848–863. 10.1063/1.1324008. 12779434

[ejsc70236-bib-0017] Dingwell, J. B. , J. P. Cusumano , P. R. Cavanagh , and D. Sternad . 2001. “Local Dynamic Stability Versus Kinematic Variability of Continuous Overground and Treadmill Walking.” Journal of Biomechanical Engineering 123, no. 1: 27–32. 10.1115/1.1336798. 11277298

[ejsc70236-bib-0018] Dingwell, J. B. , and L. C. Marin . 2006. “Kinematic Variability and Local Dynamic Stability of Upper Body Motions When Walking at Different Speeds.” Journal of Biomechanics 39, no. 3: 444–452. 10.1016/j.jbiomech.2004.12.014. 16389084

[ejsc70236-bib-0019] England, S. A. , and K. P. Granata . 2007. “The Influence of Gait Speed on Local Dynamic Stability of Walking.” Gait & Posture 25, no. 2: 172–178. 10.1016/j.gaitpost.2006.03.003. 16621565 PMC1785331

[ejsc70236-bib-0020] Fellin, R. E. , W. C. Rose , T. D. Royer , and I. S. Davis . 2010. “Comparison of Methods for Kinematic Identification of Footstrike and toe‐off During Overground and Treadmill Running.” Journal of Science and Medicine in Sport 13, no. 6: 646–650. 10.1016/J.JSAMS.2010.03.006. 20478742 PMC3266867

[ejsc70236-bib-0021] Fuller, J. T. , C. R. Bellenger , D. Thewlis , et al. 2017. “Tracking Performance Changes With Running‐Stride Variability When Athletes Are Functionally Overreached.” International Journal of Sports Physiology and Performance 12, no. 3: 357–363. 10.1123/ijspp.2015-0618. 27295717

[ejsc70236-bib-0078] Grassberger, P. , and I. Procaccia . 1983. “Measuring the Strangeness of Strange Attractors”. Physica D: Nonlinear Phenomena 9, no. 1–2: 189–208. 10.1016/0167-2789(83)90298-1.

[ejsc70236-bib-0023] Hanavan, E. P. 1964. A Mathematical Model of the Human body(AMRL‐TR‐64‐102). Aerospace Medical Research Laboratories. Wright‐Patterson Air Force Base. 14243640

[ejsc70236-bib-0024] Hausdorff, J. M. , S. L. Mitchell , R. Firtion , et al. 1997. “Altered Fractal Dynamics of Gait: Reduced Stride‐Interval Correlations With Aging and Huntington’s Disease.” Journal of Applied Physiology 82, no. 1: 262–269. 10.1152/jappl.1997.82.1.262. 9029225

[ejsc70236-bib-0025] Hoenig, T. , D. Hamacher , K.‐M. M. Braumann , A. Zech , and K. Hollander . 2019. “Analysis of Running Stability During 5000 m Running.” European Journal of Sport Science 19, no. 4: 413–421. 10.1080/17461391.2018.1519040. 30257130

[ejsc70236-bib-0026] Hollander, K. , D. Hamacher , and A. Zech . 2021. “Running Barefoot Leads to Lower Running Stability Compared to Shod Running—Results From a Randomized Controlled Study.” Scientific Reports 11, no. 1: 4376. 10.1038/s41598-021-83056-9. 33623054 PMC7902604

[ejsc70236-bib-0027] Hunter, B. , A. Greenhalgh , B. Karsten , M. Burnley , and D. Muniz‐Pumares . 2021. “A Non‐Linear Analysis of Running in the Heavy and Severe Intensity Domains.” European Journal of Applied Physiology 121, no. 5: 1297–1313. 10.1007/s00421-021-04615-6. 33580289

[ejsc70236-bib-0028] Hunter, B. , B. Karsten , A. Greenhalgh , M. Burnley , and D. Muniz‐Pumares . 2023. “The Application of Non‐Linear Methods to Quantify Changes to Movement Dynamics During Running: A Scoping Review.” Journal of Sports Sciences 41, no. 5: 481–494. 10.1080/02640414.2023.2225014. 37330658

[ejsc70236-bib-0029] James . 2004. “Considerations of Movement Variability in Biomechanics Research.” Innovative Analyses of Human Movement, edited by Stergiou, N. , 29–62. Human Kinetics.

[ejsc70236-bib-0030] Jones, A. M. , and J. H. Doust . 1996. “A 1% Treadmill Grade Most Accurately Reflects the Energetic Cost of Outdoor Running.” Journal of Sports Sciences 14, no. 4: 321–327. 10.1080/02640419608727717. 8887211

[ejsc70236-bib-0031] Jordan, K. , J. H. Challis , J. P. Cusumano , and K. M. Newell . 2009. “Stability and the Time‐Dependent Structure of Gait Variability in Walking and Running.” Human Movement Science 28, no. 1: 113–128. 10.1016/j.humov.2008.09.001. 19042050

[ejsc70236-bib-0032] Jordan, K. , J. H. Challis , and K. M. Newell . 2006. “Long Range Correlations in the Stride Interval of Running.” Gait & Posture 24, no. 1: 120–125. 10.1016/j.gaitpost.2005.08.003. 16182530

[ejsc70236-bib-0033] Jordan, K. , J. H. Challis , and K. M. Newell . 2007. “Speed Influences on the Scaling Behavior of Gait Cycle Fluctuations During Treadmill Running.” Human Movement Science 26, no. 1: 87–102. 10.1016/j.humov.2006.10.001. 17161484

[ejsc70236-bib-0034] Kadaba, M. P. , H. K. Ramakrishnan , and M. E. Wootten . 1990. “Measurement of Lower Extremity Kinematics During Level Walking.” Journal of Orthopaedic Research 8, no. 3: 383–392. 10.1002/jor.1100080310. 2324857

[ejsc70236-bib-0035] Karsten, B. , J. Baker , F. Naclerio , A. Klose , A. Bianco , and A. Nimmerichter . 2017. “Time Trials Versus Time to Exhaustion Tests: Effects on Critical Power, W′ and Oxygen Uptake Kinetics.” International Journal of Sports Physiology and Performance: 1–22. 10.1123/ijspp.2016-0761 28530476

[ejsc70236-bib-0036] Kennel, M. B. , R. Brown , and H. D. I. Abarbanel . 1992. “Determining Embedding Dimension for Phase‐Space Reconstruction Using a Geometrical Construction.” Physical Review A 45, no. 6: 3403–3411. 10.1103/PhysRevA.45.3403. 9907388

[ejsc70236-bib-0037] Lindsay, T. R. , T. D. Noakes , and S. J. Mcgregor . 2014. “Effect of Treadmill Versus Overground Running on the Structure of Variability of Stride Timing.” Perceptual and Motor Skills 118, no. 2: 331–346. 10.2466/30.26.PMS.118k18w8. 24897871

[ejsc70236-bib-0038] Look, N. , C. J. Arellano , A. M. Grabowski , W. J. McDermott , R. Kram , and E. Bradley . 2013. “Dynamic Stability of Running: The Effects of Speed and Leg Amputations on the Maximal Lyapunov Exponent.” Chaos 23, no. 4: 043131. 10.1063/1.4837095. 24387570

[ejsc70236-bib-0039] Mahaki, M. , R. Mimar , H. Sadeghi , M. Khaleghi Tazji , and M. F. Vieira . 2020. “The Effects of General Fatigue Induced by Incremental Exercise Test and Active Recovery Modes on Energy Cost, Gait Variability and Stability in Male Soccer Players.” Journal of Biomechanics 106: 109823. 10.1016/j.jbiomech.2020.109823. 32517989

[ejsc70236-bib-0040] Mann, R. , L. Malisoux , C. Nührenbörger , A. Urhausen , K. Meijer , and D. Theisen . 2015. “Association of Previous Injury and Speed With Running Style and stride‐to‐stride Fluctuations.” Scandinavian Journal of Medicine & Science in Sports 25, no. 6: e638–e645. 10.1111/sms.12397. 25557130

[ejsc70236-bib-0041] Martino, G. , Y. P. Ivanenko , A. d’Avella , et al. 2015. “Neuromuscular Adjustments of Gait Associated With Unstable Conditions.” Journal of Neurophysiology 114, no. 5: 2867–2882. 10.1152/JN.00029.2015. 26378199 PMC4737426

[ejsc70236-bib-0042] McKay, A. K. A. , T. Stellingwerff , E. S. Smith , et al. 2021. “Defining Training and Performance Caliber: A Participant Classification Framework.” International Journal of Sports Physiology and Performance 17, no. 2: 317–331. 10.1123/IJSPP.2021-0451. 34965513

[ejsc70236-bib-0043] Mehdizadeh, S. 2018. “The Largest Lyapunov Exponent of Gait in Young and Elderly Individuals: A Systematic Review.” Gait & Posture 60: 241–250. 10.1016/j.gaitpost.2017.12.016. 29304432

[ejsc70236-bib-0044] Mehdizadeh, S. , A. R. Arshi , and K. Davids . 2014. “Effect of Speed on Local Dynamic Stability of Locomotion Under Different Task Constraints in Running.” European Journal of Sport Science 14, no. 8: 791–798. 10.1080/17461391.2014.905986. 24720520

[ejsc70236-bib-0045] Mehdizadeh, S. , A. R. Arshi , and K. Davids . 2016. “Constraints on Dynamic Stability During Forward, Backward and Lateral Locomotion in Skilled Football Players.” European Journal of Sport Science 16, no. 2: 190–198. 10.1080/17461391.2014.995233. 25553807

[ejsc70236-bib-0046] Mello, R. G. T. , L. F. de Oliveira , and J. Nadal . 2010. “Effects of Maximal Oxygen Uptake Test and Prolonged Cycle Ergometer Exercise on the Quiet Standing Control.” Gait & Posture 32, no. 2: 220–225. 10.1016/j.gaitpost.2010.04.016. 20542431

[ejsc70236-bib-0047] Meyler, S. , L. Bottoms , and D. Muniz‐Pumares . 2021. “Biological and Methodological Factors Affecting VO2max Response Variability to Endurance Training and the Influence of Exercise Intensity Prescription.” Experimental Physiology 106, no. 7: 1410–1424. 10.1113/EP089565. 34036650

[ejsc70236-bib-0048] Mileti, I. , A. Serra , N. Wolf , et al. 2020. “Muscle Activation Patterns Are More Constrained and Regular in Treadmill than in Overground Human Locomotion.” Frontiers in Bioengineering and Biotechnology 8: 581619. 10.3389/FBIOE.2020.581619. 33195143 PMC7644811

[ejsc70236-bib-0049] Mo, S. , and D. H. K. K. Chow . 2018. “Stride‐To‐Stride Variability and Complexity Between Novice and Experienced Runners During a Prolonged Run at Anaerobic Threshold Speed.” Gait & Posture 64: 7–11. 10.1016/j.gaitpost.2018.05.021. 29803083

[ejsc70236-bib-0050] Murray, A. , J. H. Ryu , J. Sproule , A. P. Turner , P. Graham‐Smith , and M. Cardinale . 2017. “A Pilot Study Using Entropy as a Noninvasive Assessment of Running.” International Journal of Sports Physiology and Performance 12, no. 8: 1119–1122. 10.1123/ijspp.2016-0205. 28095075

[ejsc70236-bib-0051] Ogaya, S. , M. Suzuki , C. Yoshioka , Y. Nakamura , S. Kita , and K. Watanabe . 2021. “The Effects of Trunk Endurance Training on Running Kinematics and Its Variability in Novice Female Runners.” Sports Biomechanics 23, no. 8: 997–1008. 10.1080/14763141.2021.1906938. 33906577

[ejsc70236-bib-0052] Padulo, J. , M. Ayalon , F. A. Barbieri , et al. 2022. “Effects of Gradient and Speed on Uphill Running Gait Variability.” Sports Health 15, no. 1: 67–73. 10.1177/19417381211067721. 35343321 PMC9808836

[ejsc70236-bib-0053] Peng, C. K. , S. V. Buldyrev , S. Havlin , M. Simons , H. E. Stanley , and A. L. Goldberger . 1994. “Mosaic Organization of DNA Nucleotides.” Physical Review E 49, no. 2: 1685–1689. 10.1103/physreve.49.1685. 9961383

[ejsc70236-bib-0054] Pethick, J. , S. L. Winter , and M. Burnley . 2016. “Loss of Knee Extensor Torque Complexity During Fatiguing Isometric Muscle Contractions Occurs Exclusively Above the Critical Torque.” American Journal of Physiology—Regulatory, Integrative and Comparative Physiology 310, no. 11: R1144–R1153. 10.1152/ajpregu.00019.2016. 27101290

[ejsc70236-bib-0055] Pethick, J. , S. L. Winter , and M. Burnley . 2019. “Relationship Between Muscle Metabolic Rate and Muscle Torque Complexity During Fatiguing Intermittent Isometric Contractions in Humans.” Physiological Reports 7, no. 18. 10.14814/phy2.14240. PMC6759514 31552708

[ejsc70236-bib-0056] Phinyomark, A. , R. Larracy , and E. Scheme . 2020. “Fractal Analysis of Human Gait Variability via Stride Interval Time Series.” Frontiers in Physiology 11: 333. 10.3389/fphys.2020.00333. 32351405 PMC7174763

[ejsc70236-bib-0057] Raffalt, P. C. , J. A. Kent , S. R. Wurdeman , and N. Stergiou . 2019. “Selection Procedures for the Largest Lyapunov Exponent in Gait Biomechanics.” Annals of Biomedical Engineering 47, no. 4: 913–923. 10.1007/s10439-019-02216-1. 30701396 PMC6438190

[ejsc70236-bib-0058] Raffalt, P. C. , J. A. Kent , S. R. Wurdeman , and N. Stergiou . 2020. “To Walk or to Run—A Question of Movement Attractor Stability.” Journal of Experimental Biology 223, no. Pt 13: jeb.224113. 10.1242/jeb.224113. PMC7338268 32527966

[ejsc70236-bib-0059] Raffalt, P. C. , B. Senderling , and N. Stergiou . 2020. “Filtering Affects the Calculation of the Largest Lyapunov Exponent.” Computers in Biology and Medicine 122: 103786. 10.1016/j.compbiomed.2020.103786. 32479345

[ejsc70236-bib-0060] Rosenstein, M. T. , J. J. Collins , and C. J. De Luca . 1993. “A Practical Method for Calculating Largest Lyapunov Exponents From Small Data Sets.” Physica D: Nonlinear Phenomena 65, no. 1–2: 117–134. 10.1016/0167-2789(93)90009-p.

[ejsc70236-bib-0061] Saibene, F. , and A. E. Minetti . 2003. “Biomechanical and Physiological Aspects of Legged Locomotion in Humans.” European Journal of Applied Physiology 88, no. 4–5: 297–316. 10.1007/s00421-002-0654-9. 12527959

[ejsc70236-bib-0062] Santuz, A. , A. Ekizos , N. Eckardt , A. Kibele , and A. Arampatzis . 2018. “Challenging Human Locomotion: Stability and Modular Organisation in Unsteady Conditions.” Scientific Reports 8, no. 1: 1–13. 10.1038/s41598-018-21018-4. 29426876 PMC5807318

[ejsc70236-bib-0063] Santuz, A. , A. Ekizos , Y. Kunimasa , K. Kijima , M. Ishikawa , and A. Arampatzis . 2020. “Lower Complexity of Motor Primitives Ensures Robust Control of High‐Speed Human Locomotion.” Heliyon 6: e05377. 10.1016/j.heliyon.2020.e05377. 33163662 PMC7610320

[ejsc70236-bib-0064] Schütte, K. H. , E. A. Maas , V. Exadaktylos , D. Berckmans , R. E. Venter , and B. Vanwanseele . 2015. “Wireless Tri‐axial Trunk Accelerometry Detects Deviations in Dynamic Center of Mass Motion due to Running‐Induced Fatigue.” PLoS One 10, no. 10: 1–12. 10.1371/journal.pone.0141957. PMC4627812 26517261

[ejsc70236-bib-0065] Stergiou, N. , ed. 2018. Nonlinear Analysis for Human Movement Variability. CRC Press. https://www.scopus.com/inward/record.uri?eid=2‐s2.0‐84980346598&partnerID=40&md5=5773ae197a2de264e3863ed071a7019a.

[ejsc70236-bib-0066] Stergiou, N. , and L. M. Decker . 2011. “Human Movement Variability, Nonlinear Dynamics, and Pathology: Is There a Connection?” Human Movement Science 30, no. 5: 869–888. 10.1016/j.humov.2011.06.002. 21802756 PMC3183280

[ejsc70236-bib-0067] Strongman, C. , and A. Morrison . 2020. “A Scoping Review of Non‐linear Analysis Approaches Measuring Variability in Gait due to Lower Body Injury or Dysfunction.” Human Movement Science 69: 102562. 10.1016/j.humov.2019.102562. 31989953

[ejsc70236-bib-0068] Strongman, C. , and A. Morrison . 2021. “Evaluating Dynamic Similarity of Fixed, Self‐Selected and Anatomically Scaled Speeds in Non‐Linear Analysis of Gait During Treadmill Running.” Human Movement Science 76: 102768. 10.1016/j.humov.2021.102768. 33556908

[ejsc70236-bib-0069] Valencia, O. , A. Weinstein , R. Salas , R. Guzmán‐Venegas , M. Arvanitidis , and E. Martinez‐Valdes . 2023. “Temporal Differences in the Myoelectric Activity of Lower Limb Muscles During Rearfoot and Forefoot Running: A Statistical Parametric Mapping Approach.” European Journal of Sport Science 23, no. 6: 983–991. 10.1080/17461391.2022.2081094. 35593659

[ejsc70236-bib-0070] Vanhatalo, A. , M. I. Black , F. J. DiMenna , et al. 2016. “The Mechanistic Bases of the Power–Time Relationship: Muscle Metabolic Responses and Relationships to Muscle Fibre Type.” Journal of Physiology 594, no. 15: 4407–4423. 10.1113/jp271879. 26940850 PMC4967754

[ejsc70236-bib-0071] Vieira, M. F. , G. S. de Sá e Souza , G. C. Lehnen , F. B. Rodrigues , and A. O. Andrade . 2016. “Effects of General Fatigue Induced by Incremental Maximal Exercise Test on Gait Stability and Variability of Healthy Young Subjects.” Journal of Electromyography and Kinesiology 30: 161–167. 10.1016/j.jelekin.2016.07.007. 27451361

[ejsc70236-bib-0072] Walsh, G. S. 2021. “Dynamics of Modular Neuromotor Control of Walking and Running During Single and Dual Task Conditions.” Neuroscience 465: 1–10. 10.1016/J.NEUROSCIENCE.2021.04.004. 33887387

[ejsc70236-bib-0073] Willer, J. , S. J. Allen , R. J. Burden , and J. P. Folland . 2024. “How Humans Run Faster: The Neuromechanical Contributions of Functional Muscle Groups to Running at Different Speeds.” Scandinavian Journal of Medicine & Science in Sports 34, no. 8: e14690. 10.1111/sms.14690. 39049546

[ejsc70236-bib-0074] Wilson, J. M. , and E. P. Flanagan . 2008. “The Role of Elastic Energy in Activities With High Force and Power Requirements: A Brief Review.” Journal of Strength & Conditioning Research 22, no. 5: 1705–1715. 10.1519/JSC.0B013E31817AE4A7. 18714212

[ejsc70236-bib-0075] Winter, L. , P. Taylor , C. Bellenger , P. Grimshaw , and R. G. Crowther . 2023. “The Application of the Lyapunov Exponent to Analyse Human Performance: A Systematic Review”. Journal of Sports Sciences 41, no. 22: 1994–2013. 10.1080/02640414.2024.2308441. 38326239

[ejsc70236-bib-0076] Yentes, J. M. , N. Hunt , K. K. Schmid , J. P. Kaipust , D. McGrath , and N. Stergiou . 2013. “The Appropriate Use of Approximate Entropy and Sample Entropy With Short Data Sets.” Annals of Biomedical Engineering 41, no. 2: 349–365. 10.1007/s10439-012-0668-3. 23064819 PMC6549512

[ejsc70236-bib-0077] Zeni, J. A. , J. G. Richards , and J. S. Higginson . 2008. “Two Simple Methods for Determining Gait Events During Treadmill and Overground Walking Using Kinematic Data.” Gait & Posture 27, no. 4: 710–714. 10.1016/J.GAITPOST.2007.07.007. 17723303 PMC2384115

